# Books Reviewed

**Published:** 1867-06

**Authors:** 


### Books Reviewed.

Obstetrics: The Science and the Art. By Charles D. Meigs, M. D. Philadelphia: Henry C. Lea, 1867.

The readers of this Journal are too well acquainted with the character and standing of this work to have any extended notice, interesting or even proper. We notice by the preface, that the author has carefully revised every paragraph, with the view of leaving his work to the profession as perfect and complete as possible, thinking that this is probably the last occasion he will ever have of making it a better book for instruction than before. In turning over the pages of this last and most perfect edition, we are attracted by the manner he writes upon albuminuria. He says: "What now is albuminuria; for that is the question? Is albuminuria a disease, or is it a symptom? Such is the question put by Gourbeyre. Is albuminuria a leak from a disordered and imperfect kidney of one of the important materials of the blood? Is albuminuria a result of modified power of hæmatocis, and is it not rather a disease of the blood-vessel, the endangium, than of Bowman's capsules or Malphighi's corpuscles? Goubeyre quotes Frerichs to show that in 202 *post mortem* examinations of morbus brightii, the lesions of the kidneys were more numerous than any others; there was heart disease 99 times; emphysema, 77; diseased liver, 46; (22 cirrhosis) spleen, 50. Dr. Gourbeyre insists that morbus brightii, commonly called albuminuria, and albuminuric nephritis, is nothing more or less than disalbuminuriation of the blood, and that disalbuminuriation of the blood may take place in persons whose health is perfect as far as the kidney is concerned, and in whose urine neither tubuli-casts nor the least trace of albumen can be detected. What then, I repeat, is albuminuria of gestation? Is it not a vitiated state of the blood dependent on morbid changes superinduced by pregnancy in the blood membrane; and is it anything else than a symptom at once, and a cause of hydræmia? Is it not in many cases developed during the labor in consequence of the violence of the circulation? The very strongest advocate of the doctrine of uræmic intoxication as causative of eclampsia, Dr. C. Braun, says, that there is no constant relation of the quantity of albumen in the urine to the period of labor in which the convulsions break out, but the amount of albumen does augment in the ratio of the repetitions of the eclampsic paroxysms. To me it appears that this admission strengthens the opinion that child-bed fits depend not so much on the qualities of the blood, as on its impetus in circulation, and that its morbid qualities are rather results than causes; although to be sure the morbid results do become causes in their turn."

This subject is considered at length and is interesting in many respects; the quotation thus far, has been made with no other view than to indicate the author's views upon an important pathological point. The work comprises a complete treatise upon obstetrics, and stands high among the very first works upon this subject. Its style is unexceptionable, and shows it to be the product of high mental discipline, careful observation and ripe experience. The opinions of the author are entitled to great respect, and this book, in its fifth and last edition, will remain a standing authority upon all subjects in the department of obstetrics.

The publisher, Henry C. Lea, of Philadelphia, is entitled to the thanks of the profession, for the neat, substantial and ornamental style of his medical publications in general; this one in particular.

For sale in Buffalo by Theodore Butler.

---

Practical Dissections. By Richard M. Hodges, M. D., formerly Demonstrator of Harvard University. Second edition, thoroughly revised. Philadelphia: Henry C. Lea, 1867.

After a careful examination of the "Practical Dissections," we can fully commend it to the favorable consideration of all engaged in the pursuit of practical anatomy. Their design is not intended to occupy the place of a treatise on anatomy, but simply as "a practical guide in the ordinary dissections of the medical student, describing on the same page, and in connection, the muscles, nerves, arteries, veins or other structures which are conjointly exposed in dissecting any one of the parts into which the dead subject is usually divided." An experience of more than seven years in demonstrating, has enabled the author to arrange his work in such a manner as will be found most convenient. Part first, comprises the anatomy of the head and neck, embraced in fourteen dissections. Part second, comprises the anatomy of the upper extremities, thorax and back, divided into ten dissections; and part third, embracing the anatomy of the abdomen and lower extremities, with fourteen dissections. The division of each part into "dissections" is intended to map out a day's work, thus affording to the student an opportunity to prepare each succeeding day's work. Besides describing the peculiarities of the anatomy of the fœtus and the surgical anatomy of the arteries, the author has given some general rules to be observed in dissecting, which, if followed, will greatly add to the neatness of the dissections, as also to the personal comfort of the dissector.

---

The Half-Yearly Abstract of the Medical Sciences; being an analytical and critical digest of the principal British and Continental Medical works, published in the preceding six months. Vol. 44, July–December, 1866. Philadelphia: Henry C. Lea, 1867.

The medical profession is too well acquainted with the "Half-Yearly Abstract" to require any extensive notice on our part. The present volume, like its predecessors, is full of interest, embodying within its pages the cream of foreign medical literature, and no practitioner desiring to remain acquainted with foreign literature can well afford to be without this work.

---

What effect has the Meat or Milk from Diseased Animals on the Public Health? By Samuel R. Percy, M. D., Professor of Materia Medica New York Medical College; Physician to the Jews' Hospital, etc., 1866.

Questions ot grave importance to the public health are involved in the consideration of this subject, and Dr. Percy by earnest and careful investigation has furnished to the medical profession a clew to the cause of many diseases heretofore not recognized. We trust that he will continue to labor in this almost untrodden field of investigation, and that medical societies and health commissioners will extend to him such support as the importance of the subject demands.

Diphtheria: A prize essay. By E. S. Gaillard, M. D., Richmond, Virginia, 1867.

This monograph (a reprint from the Richmond Medical Journal) is designed chiefly for the elucidation and establishment of the pathology of the disease under consideration, and with this view, the author has consulted the best medical authorities upon this subject both in America and Europe. The arrangement, in a tabular form, of the differences between the pathology of diphtheria and that of the diseases with which it is most frequently confounded, namely: pharyngitis, muguet, aphthous, inflammation of the mouth, croup and scarlet fever, possesses advantages which are not found in works of a similar character.

The indications for treatment the author considers both as constitutional and local. Under the former he strongly advocates the sustaining plan, while general and local depletion, vesiccatories, sinapisms, etc., should be entirely discarded. In the local treatment much benefit has been derived from the following preparation, to be applied to the exudation:

Aquæ Fluv. grs. xxxii,

Ferro Perchlorid,

Acid Citric,

Acid Hydrochlo, } aa grs. iv.

The forcible tearing away of the membrane the author considers as worse than useless.

---

Prize Essay—Digitalin. Its chemical, physiological and therapeutic action. An essay to which was awarded a prize by the American Medical Association, May, 1866. By Samuel R. Percy, M. D., Professor of Materia Medica New York Medical College, etc., etc.

The adjudgment of a prize to this essay, by the American Medical Association is a sufficient guarantee of its value, and all that remains for us to do is to simply present its arrangement for the benefit of those of our readers who may not possess the Transactions for 1866. The author presents his subject in four sections. Section first, treating of its chemical history; process of obtaining the alkaloid; other principles contained in it; its physical properties; chemical tests and reagents. Section second, discusses its physiological action upon animals and man: Section third, considers the therapeutics of digitalin; cumulative action; effects in poisonous doses. Section fourth, treats of the modus operandi.

---

Researches upon Spurious Vaccination or the Abnormal Phenomena accompanying and following Vaccination in the Confederate Army, during the recent American Civil War, 1861-1865. By Joseph Jones, M. D., Professor of Physiology and Pathology in the Medical Department of the University of Nashville, Tenn.

Never before has the subject of spurious vaccination been treated so comprehensively in all its various bearings as by the author. The influences of scorbutus, syphilis and skin diseases upon vaccine are ably discussed, and his conclusions, supported as they are by the observations of the best medical men, carry with them an overpowering weight. The perusal of this monograph will be found highly interesting and instructive.

The American Conflict: A history of the Great Rebellion in the United States of America, 1860–1865: Its causes and incidents, and results. Intended to exhibit especially, its moral and political phases, with the drift and progress of American opinion respecting human slavery, from 1776, to the close of the War for the Union. By Horace Greely. Illustrated by portraits on steel, of generals, statesmen and other eminent men; views of places of historical interest, maps, diagrams of battle-fields, naval actions, etc., from official sources. Vols. I & II.

From the very day of our national birth, two antagonistic principles have been contending in the country for the mastery. Freedom and Slavery; the slaveholding power inaugurating a desperate struggle to retain, by force of arms, that which, by the natural operation of the ballot-box, they were slowly but surely losing. Beginning at the very beginning of the causes of the late civil war, the author has spared no pains in the collection of material facts, and to arrange them so as to embody "the more essential documents or part of documents, illustrative of these facts, that the attentive, intelligent reader may learn from this work, not only what were the leading incidents in our civil war, but its causes, incitements, and the inev. itable sequence, whereby ideas proved the germ of events:" Never before has the political history of our country been placed before us in such a dispassionate and interesting form; the constant aim of the author being to avoid and discard party feelings. In reference to the description of battles, the course of the writer has been to omit those minute and complicated details which can only prove wearisome to the general reader. And the sources of information from which these descriptions found their existence, have been authentic reports from the War Department; the official statements of Commandants on the battle-field, and many graphic descriptions from eye-witnesses.

Lewis A. Filbert, 133 Elm street, agent for Buffalo.

---

A case of luxation of femur into Ischiatic notch, of nine months standing, reduced by manipulation. By Lewis A. Sayre, M. D., Surgeon of Bellevue Hospital; Professor of Orthopœdic Surgery, etc., etc.

This monograph describes a very interesting and instructive case of luxation of the femur, the result of a gun-shot injury. Five months after sustaining the injury, and while the patient changed his position in bed, the head of the bone glided out of its socket. Several attempts at reduction having been made, all of which proved unsuccessful, the patient placed himself under the professional care of the writer; who, nine months from the time of the luxations, made an attempt at reduction. Having by a rectum examination felt the gliding of the head of the bone, he determined to reduce the luxation after the method of Dr. Reid, which proved success. ful, the parallelism of the limb being maintained by means of extension by weight and pully. Ten months subsequently the patient states that he can step in any direction, as easily as with the sound limb.

---

An inquiry into Modern Anæsthesia. By Honorable Truman Smith.

This work comprises an argument for the claims of Horace Wells, to the discovery of anæsthesia, and contains all the facts connected with it, and Dr. Morton's claim as discoverer. The author says: "In 1844, Horace Wells gave the world his wonderful discovery that surgery could be divested of pain—a discovery pregnant with untold value to the world, but of almost unmingled woe and sorrow to himself and his afflicted family. Often have I heard his widow declare that this great boon to the world, "had been to her and her family an unsupportable evil," for it cost the life of her husband, and substituted the "*res angusti domi*" in place of a lucrative profession and a happy home. Knowing Wells intimately, living beneath the same roof at the time when he went to Boston to announce his discovery, and in almost daily communication with him during the whole period between the birth of his great thought and the hour when his dead body, a sacrifice to his zeal and love of truth, was borne from my own door to its last resting place, I can, and do, before high Heaven, that to Horace Wells only, belongs the honor of giving to the world a discovery of which has played a more important part, as respects surgery, than any other ever made, unless we except Harvey's of the circulation of the blood. The full value of his discovery is not yet known; after-ages will make new applications and further improvements." This quotation shows the object of the work, and without expressing personal opinions, we must heartily commend its perusal to every one interested to know all the facts in this matter so long in controversy.

---

The American Naturalist. A Popular Illustrated Magazine of Natural History. Salem: Essex Institute.

This valuable magazine is the only one in the country devoted to Natural History, thus supplying a deficiency which had heretofore been existing in American journalism. The Naturalist, we have no doubt, will certainly meet the approbation of those interested in the study af natural sciences, and prove a valuable companion to tourists, as reliable information relating to many interesting facts in natural history is placed before them. The publishers have spared no labor nor money to present a work which in general appearance is unsurpassed, and we trust their efforts will be liberally sustained by the public. Price, $2.00.

